# Zebrafish Vestigial Like Family Member 4b Is Required for Valvulogenesis Through Sequestration of Transcription Factor Myocyte Enhancer Factor 2c

**DOI:** 10.3389/fcell.2019.00277

**Published:** 2019-11-12

**Authors:** Chang Xue, Xiaohui Liu, Bin Wen, Ruimeng Yang, Shuo Gao, Jiong Tao, Jun Zhou

**Affiliations:** ^1^CNRS-LIA Hematology and Cancer, Sino-French Research Center for Life Sciences and Genomics, State Key Laboratory of Medical Genomics, RuiJin Hospital, Shanghai Jiao Tong University School of Medicine, Shanghai, China; ^2^Prenatal Diagnosis Center, Shanghai Jiao Tong University Affiliated First People’s Hospital, Shanghai, China

**Keywords:** *vgll4b*, valvulogenesis, zebrafish, Mef2c, *klf2a*, endocardium, *tead*

## Abstract

A variety of cardiac transcription factors/cofactors, signaling pathways, and downstream structural genes integrate to form the regulatory hierarchies to ensure proper cardiogenesis in vertebrate. Major interaction proteins of the transcription cofactor vestigial like family member 4 (VGLL4) include myocyte enhancer factor 2 (MEF2) and TEA domain transcription factors (TEAD), both of which play important roles in embryonic cardiac development and in adulthood. In this study, we identified that the deficiency of zebrafish *vgll4b* paralog, a unique family member expressed in developing heart, led to an impaired valve development. Mechanistically, in *vgll4b* mutant embryos the disruption of Vgll4b-Mef2c complex, rather than that of Vgll4b-Tead complex, resulted in an aberrant expression of *krüppel-like factor 2a* (*klf2a*) in endocardium. Such misexpression of *klf2a* eventually evoked the valvulogenesis defects. Our findings suggest that zebrafish Vgll4b plays an important role in modulating the transcription activity of Mef2c on *klf2a* during valve development in a blood-flow-independent manner.

## Introduction

Four TONDU (TDU) domain-containing proteins, named VGLL1 to VGLL4 exist in mammals. As transcription cofactor, VGLL proteins do not contain a DNA binding domain (DBD), their transcriptional activities could only be mediated through the TDU domain which interacts with different transcription factors such as TEADs (also named TEFs) and MEF2s (Chen et al., 2004a). Structurally, unlike VGLL1 to 3 which only possess one TDU domain, there are two tandem TDU domains located in VGLL4. *VGLL* genes play different roles in various physiological processes. For example, VGLL2 acts as a cofactor of TEAD1 during skeletal muscle development (Maeda et al., 2002; Chen et al., 2004b; Honda et al., 2017) while VGLL4 functions as a positive regulator in survival of human embryonic stem cells (hESCs) (Tajonar et al., 2013). Numerous studies indicate that *VGLL* genes participate in tumorigenesis. *VGLL1* is highly expressed in basal-like breast cancer and promotes cell proliferation (Castilla et al., 2014). Similarly, *VGLL3* is overexpressed in a subset of soft tissue sarcomas (Hélias-Rodzewicz et al., 2009). Interestingly, the expression of *VGLL3* was absent in high-grade serous ovarian carcinomas (HGSC), suggesting *VGLL3* might also be involved in tumor suppressor pathways (Gambaro et al., 2013). Among all of the family members, *VGLL4* is the most frequently tumorigenesis-related one. Decreased expression level of *VGLL4* has been observed in multiple types of tumors including gastric cancer (Jiao et al., 2014), lung cancer (Zhang et al., 2014), breast cancer (Zhang et al., 2017), colorectal cancer (Jiao et al., 2017), and esophageal squamous cell carcinoma (ESCC) (Jiang et al., 2015). The Hippo signaling pathway is highly conserved in mammals and implicated in regulation of cell growth and organ size. The transcription coactivator YAP is one major downstream target of Hippo pathway which binds to transcription factors to activate gene expression and regulate cell growth or death (Kim et al., 2019). In the malignancies mentioned above the balance between TEAD-VGLL4 and TEAD-YAP complexes is disrupted (Jiao et al., 2014; Zhang et al., 2014, 2017), thus VGLL4 is regarded as a tumor suppressor by negatively regulating the Hippo pathway to inhibit cell proliferation.

Since the presence of an extra TDU domain, VGLL4 is believed to be functionally different with other VGLLs. Actually their expression patterns are quite different. VGLL1 and VGLL3 are enriched in placenta, whereas VGLL2 is detected only in skeletal muscle (Maeda et al., 2002). *VGLL4* is the unique *VGLL* gene expressed in heart (Chen et al., 2004a), moreover, it has been reported that from the post-endothelial to mesenchyme transition (EMT) stage to adult stage murine *Vgll4* is notably expressed in heart valve endothelial and interstitial cells, and endothelial loss of *Vgll4* results in valve malformation (Yu et al., 2019). Mechanistic studies reveal that VGLL4 competes with YAP for TEADs binding to regulate valvulogenesis (Yu et al., 2019).

The heart is the first functional organ in vertebrate embryo (Moorman et al., 2003). Heart development begins with the formation of the primitive heart tube, that loops and septates into four chambers and paired arterial trunks to form the mature heart in human (Moorman et al., 2003). During heart development the formation of valve is a complicated process. Defective valvulogenesis generally leads to impaired cardiac function and congenital heart diseases (CHD) (Combs and Yutzey, 2009). In addition to mouse, xenopus and chick, zebrafish has emerged as a powerful model organism in genetic studies of cardiac development in recent decades. Unlike human counterpart, three *vgll4* paralog genes named *vgll4a*, *vgll4b* and *vgll4l* exist in zebrafish genome. We have demonstrated in our previous study that only *vgll4b*, but not *vgll4a* and *vgll4l*, is expressed in the developing heart (Xue et al., 2018). Except for TEADs, MEF2 family members are also major interactants of VGLL4 (Chen et al., 2004a), which are enriched in heart and play a variety of functions during embryonic cardiac development and in adulthood (Chen et al., 1994; Naya et al., 2002; Durham et al., 2006; Huang et al., 2006; Ewen et al., 2011; Lockhart et al., 2013; Dong et al., 2017). In the current work we took advantage of a knockout line to provide *in vivo* evidence that the zebrafish *vgll4b* paralog is indispensable for normal valvulogenesis, of which the mechanism differs from that in mice. Upon loss of Vgll4b, the Vgll4b-Mef2c complex, rather than Vgll4b-Tead complex, is disrupted in endocardium. The released Mef2c in turns aberrantly activates *klf2a*, which eventually evokes the defective valvulogenesis.

## Materials and Methods

### Zebrafish Maintenance and Mutant Generation

Zebrafish were raised, bred, and staged according to standard protocols (Kimmel et al., 1995). All animal works were strictly conducted following the guidelines of the Animal Care and Use Committee of Shanghai Jiao Tong University. For CRISPR/Cas9 system mediated *vgll4b* knockout zebrafish generation, guide RNA targeting exon1 of *vgll4b* was designed using an online tool ZiFiT Targeter software^1^, which was synthesized by cloning the annealed oligonucleotides into the sgRNA transcription vector. Cas9 mRNA and sgRNA were co-injected into one-cell stage zebrafish embryos. The injected F0 founder embryos were raised to adulthood and then outcrossed with wild type zebrafish. F1 embryos carrying potential indel mutations were raised to adulthood. Then PCR amplification and sequencing were performed on genomic DNA isolated from tail clips of F1 zebrafish to identify mutants.

### Plasmid Construction

Zebrafish *vgll4b*, *mef2cb*, *tead*, *sumo1*, *klf2a* genes and their serial mutants were cloned into PCS2^+^ vector. *Vgll4b* ΔTDU1 and ΔTDU2 mutants were constructed by PCR-mediated deletion of *vgll4b* plasmid. Primers used were listed in Table 1. Tol2-plasmid was constructed by insertion of zebrafish *mef2cb* DN or *klf2a* DN under *cmlc2* promoter (0.9 k) or *flk1* promoter (6.4 k) (Huang et al., 2003; Jin, 2005). Transgenes were transiently expressed by co-injecting 80 pg of Tol2-plasmid and 120 pg of Tol2 transpose mRNA at one-cell stage.

**TABLE 1:** Primers used in plasmid construction.

*vgll4b*	5′CGGAATTCATGCTTTTTACCAAAATGGAC 3′	5′CGCTCGAGTCAAGACACCAGGGACGGGG 3′
*vgll4b*ΔTDU1	5′CGAACACCGCCTGTAAGGAGCCCGAGCCCGTC 3′	5′GCTCGGGCTCCTTACAGGCGGTGTTCGAGTTG 3′
*vgll4b*ΔTDU2	5′CAAACTCCGTGTCCATCCTGCAGATCAAGGC 3′	5′CTTGATCTGCAGGATGGACACGGAGTTTGTG 3′
*mef2cb*	5′CCGAATTCATGGGGAGAAAAAAGATTCA 3′	5′CGCTCGAGTCATGTGGCCCACCCTTCCGA 3′
*mef2cb* DN	5′CCGAATTCATGGGGAGAAAAAAGATTCA 3′	5′CGCTCGAGTCAGGTCTCCACTATGTCCG 3′
*mef2cb* DN R3T	5′CCGAATTCATGGGGACAAAAAAGATTCA 3′	5′CGCTCGAGTCAGGTCTCCACTATGTCCG 3′
*sumo1*	5′CCGCTCGAGTCTGACCAGGAGGCAAAACC 3′	5′GCTACGTACTACGTTTGTTCCTGATAAAC 3′
*tead1a* DN	5′CCGGAATTCGATCCCAGCAGCTGGAGC 3′	5′CCGCTCGAGTTAATAAGCTGCGATTGCAGTGG 3′
*tead3b* DN	5′CCGGAATTCGACGGAGATGCAGAGGGCGTG 3′	5′CCGCTCGAGTTACACGGGCACTGCTGTGGCCAC 3′
*klf2a*	5′CCGGAATTCGCTTTGAGTGGAACGATTTTAC 3′	5′CCGCTCGAGCTACATATGACGTTTCATATG 3′
*klf2a* DN	5′CCGGAATTCGCCAAACCAAAGAGGGGGCGC 3′	5′CCGCTCGAGCTACATATGACGTTTCATATG 3′

### Whole-Mount mRNA *in situ* Hybridization (WISH)

Digoxigenin-labeled RNA probes were transcribed with T7, T3 or SP6 polymerase (Ambion, Life Technologies, United States). WISH was performed as described previously (Thisse and Thisse, 2008). The probes labeled by digoxigenin were detected using alkaline phosphatase coupled anti-digoxigenin Fab fragment antibody (Roche) with 5-bromo-4-chloro-3-indolyl-phosphate nitro blue tetrazolium staining (Vector Laboratories, Burlingame, CA, United States).

### Morpholinos and mRNA Synthesis for Microinjection

Zebrafish *vgll4b* (5′ ACAGGTCCATTTTGGTAAAAAGCAT 3′) morpholino oligonucleotides (MO) targeting the transcriptional initiation ATG of *vgll4b* was designed and purchased from Gene Tools. Full-length capped mRNA samples were all synthesized from linearized plasmids using the mMessage mMachine SP6 kit (Invitrogen, Thermo Fisher, United States). Microinjection concentration of mRNA was between 50 and 200 ng/μl and 2 nl of mRNA was injected at one-cell stage embryos. All injections were performed with a Harvard Apparatus micro-injector.

### Heart Rates Measurement and Ventricular Contractility Analysis

Zebrafish embryos were incubated at 28.5°C (VWR Scientific incubator). Cardiac function of sibling WT control and *vgll4b* mutants were quantitatively assayed by the Optical Heartbeat analysis under an Olympus IX71 microscope with HC Image software. The lengths of ventricles in diastolic and systolic conditions were measured to calculate the ventricular shortening fraction (VSF). Values are presented as mean ± SD. VSF = (short axis of ventricle in end-diastole − short axis of ventricle in end-systole)/short axis of ventricle in end-diastole.

### Immunofluorescence

Hearts were harvested manually from the embryos at 52 hpf and fixed with 4% paraformaldehyde (PFA) in PBS overnight at 4°C, followed by the permeabilization with PBS containing 0.1% Tween-20 and 0.5% Triton-X 100 for 10 min. Then the hearts were blocked in blocking buffer with 1% BSA and 10% normal goat serum for 3 h at room temperature. Mouse anti-Alcam antibody (Developmental Studies Hybridoma Bank, United States) was added in the blocking buffer and incubated for 16 h at 4°C. Goat anti-mouse Alexa-488 secondary antibody (Thermo Fisher Scientific, United States) was added in blocking buffer after thorough washing and incubated overnight at 4°C. After washing with PBS, all the samples were stained with 100 nM Tetramethylrhodamine (TRITC) labeled phalloidin solution (Solarbio, Beijing, China) containing 1% BSA. AVC cells were recognized based on their characteristic cuboidal morphologies. Images were taken using an Olympus FV1000 scanning confocal microscope. The confocal images were captured with an UPLSAPO 40 × objective.

### Quantitative RT-PCR

Quantitative PCR was carried out using a SYBR Green Real-Time PCR Master Mix (Toyobo, Osaka, Japan) with an ABI 7900HT real-time PCR machine. Three duplicated experiments were made independently for each group, β*-actin* served as the internal control. The final results were expressed as the means ± SD. All data were analyzed with GraphPad Prism software (GraphPad Software, La Jolla, CA, United States). The primers used were listed in Table 2.

**TABLE 2:** Primers used in RT-qPCR.

*klf2a*	5′CTCACTTGAAGGCTCATCAC 3′	5′GTGACGGGTCAATTCATCAG 3′
*notch1b*	5′GAATGCATCTTTTCTTCGTG 3′	5′CGTCTGCAGTTGGTTCACAT 3′

### Cell Culture and Luciferase Reporter Assay

HEK293T cells were maintained in DMEM (Life technologies, Grand Island, NY, United States) with 10% Fetal Bovine Serum (Life technologies, Grand Island, NY, United States). Plasmid transfection was carried out with Effectene Transfection Reagent (QIAGEN) according to manufacturer’s instruction. For the luciferase reporter assay, cells were harvested 48 h after transfection and analyzed using the Dual Luciferase Reporter Assay Kit (Promega, Maddison, WI, United States), according to the manufacturer’s protocols.

### Statistical Analysis

Data were presented as mean ± standard deviation (SD). For comparison of two means, statistical significance was evaluated by unpaired Student’s *t*-test. For multiple comparisons, one-way analysis of variance (ANOVA) test was used (SPSS 17.0 software, IBM, Chicago, IL, United States). Differences were considered to be significant at *P* < 0.05.

### Study Approval

The animal protocol listed above has been reviewed and approved by the Animal Ethical and Welfare Committee, Rui-Jin Hospital, Shanghai Jiao Tong University School of Medicine, Shanghai, China.

## Results

### Generation of a *vgll4b*-Deficient Zebrafish Line

**FIGURE 1:**
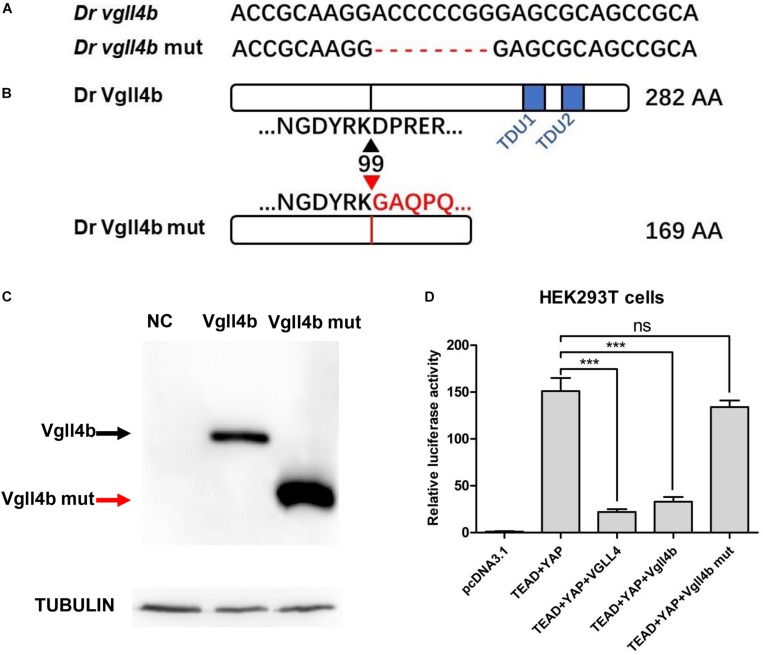
The establishment of a zebrafish *vgll4b* knockout line. **(A)** Schematic representation of Cas9 target site in the first exon of zebrafish *vgll4b*. Dr: Danio rerio. The deleted nucleotides in the mutant gene are marked by hyphens. **(B)** Schematic representation of wild type (282 amino acids) and mutant Vgll4b proteins (169 amino acids). The site where the frameshift was introduced is marked by triangles. **(C)** Western blot analysis of HA-tagged wild type and mutant Vgll4b proteins. NC: non-specific control. **(D)** Luciferase analysis of HEK293T cells transfected with TEAD1, YAP, VGLL4, and wild type or mutant Vgll4b expressing plasmids. Luciferase activity was normalized to empty vector pcDNA3.1 which was set to 1.0. Error bars represent ± SD of at least three replicates. *p* values are denoted by asterisks. ^∗∗∗^*P* < 0.001 (ANOVA test).

Zebrafish *vgll4b* gene shares high homology with its human counterpart (Xue et al., 2018). Among all of the three *vgll4* paralogs, *vgll4b* is the only one expressed in the developing heart (Xue et al., 2018). To address the role of *vgll4b* in cardiogenesis, a *vgll4b* mutant line was generated using CRISPR/Cas9 technology. Eight nucleotides were deleted, which led to a truncated form of Vgll4b without TDU domains (Figures 1A,B). The mutant *vgll4b* gene was amplified by RT-PCR from the homozygous mutant, then cloned into an HA tagged expressing vector and transfected into HEK293T cells. As anticipated, a short protein with the predicted molecular weight was detected by western blot analysis (Figure 1C). The function of the Vgll4b mutant was assessed by a dual luciferase reporter assay performed in HEK293T cells expressing TEAD, YAP, as well as wild type or the truncated Vgll4b mutant constructs. Since VGLL4 can negatively regulate the TEAD-YAP pathway, the results showed that the expression of either human *VGLL4* or zebrafish *vgll4b* could pronouncedly reduce the TEAD-YAP activity on a *TEAD* responsive reporter, whereas the zebrafish *vgll4b* mutant lacking two TDU domains could not, thus confirming the efficacy of the established mutant line (Figure 1D).

### *Vgll4b*-Deficient Zebrafish Embryos Display an Impaired Heart Valve Development

**FIGURE 2:**
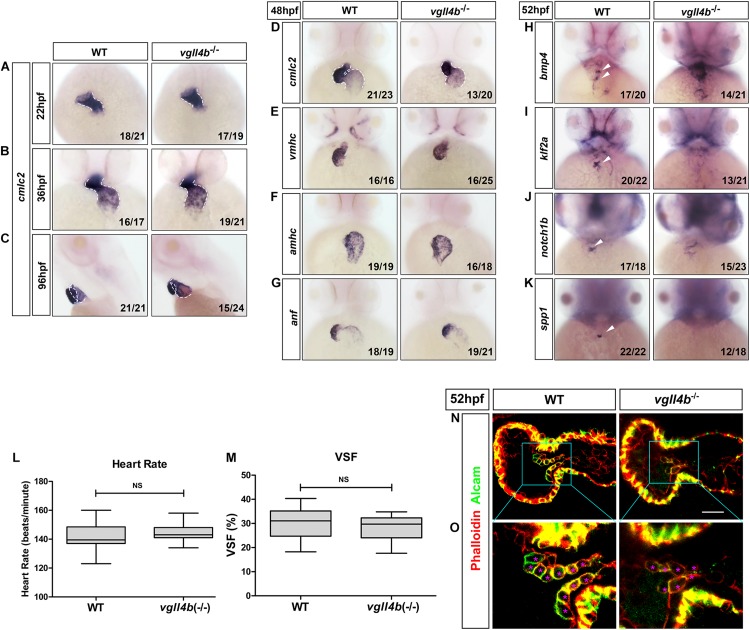
Deficiency of zebrafish *vgll4b* leads to impaired valvulogenesis. **(A–D)** WISH analysis of *cmcl2* expression in zebrafish embryos at 22, 36, 48, and 96 hpf, a defective S shaped heart looping was clearly observed from 36 hpf. White dashed lines indicate the heart morphology outlined by *cmlc2* expression. Lateral view, **(C)**. **(E–K)** WISH analysis of *vmhc*, *amhc*, *anf* at 48 hpf, as well as *bmp4*, *notch1b*, *klf2a*, *spp1* at 52 hpf. **(L,M)** Optical Heartbeat analysis of cardiac function. Comparison of the heart rate at 48 hpf **(L)** and ventricular shortening fraction at 52∼54 hfp **(M)**. **(N,O)** Representative single confocal z-section images showing the endocardial cushion cells expressing Alcam (asterisks) of wild type sibling and *vgll4b*^–/–^ embryos at 52 hpf. Scale bar: 10 μm.

As in other vertebrates, the heart in zebrafish is the first organ to form and function. The linear heart tube (LHT) forms and rhythmically contracts by 24 h post fertilization (hpf). Looping and ballooning occur at ∼36 hpf, and LHT transforms into one atrium and one ventricle by 48 hpf (Glickman and Yelon, 2002). Each chamber is composed of two tissue layers: the inner endothelial endocardium and outer muscular myocardium (Keegan, 2004). To elucidate the role of *vgll4b* in zebrafish heart development, firstly, a series of cardiomyocyte markers were examined by whole-mount mRNA *in situ* hybridization (WISH). While *cmlc2* (cardiac myosin light chain 2) marks pan myocardial cells, the two chambers are marked by two distinct myosin genes *vmhc* (ventricular myosin heavy chain) and *amhc* (atrial myosin heavy chain) (Chen et al., 1996; Yelon et al., 1999; Yelon, 2001; Berdougo, 2003). Tracking heart formation from 22 to 36 hpf, *cmlc2* staining indicated that the primary LHT and chambers formed normally in *vgll4b-*deficient embryos (Figures 2A,B), whereas a slight defective S shaped heart looping compared with sibling embryos was observed, which lasted to 96 hpf (Figures 2C,D). At 48 hpf, *cmlc2*, *vmhc* and *amhc* showed generally regular temporo-spatial expressions in *vgll4b* mutants (Figures 2D–F). Physiologically, the expression of *anf* (natriuretic peptide type A) is confined to the outer curvature (OC) of the atrium and ventricle (Auman et al., 2007), such regionalized expression of *anf* was still kept in *vgll4b-*deficient embryos, further indicating that the specification of chambers occurred independently of *vgll4b* (Figure 2G). The myocardium contracts to drive circulation. To evaluate the function of the myocardium, we assessed the heart rate (HR) and cardiac contractile function by VSF measurements (Figures 2L,M). The results showed that both HR and VSF were comparable between siblings and *vgll4b* mutants. Moreover, visual inspection analysis showed a normal blood circulation (*n* > 100 embryos analyzed). These phenomena suggest that the myocardium still functions normally upon loss of *vgll4b*.

The formation of the valves is a crucial event during cardiogenesis. The valvulogenesis process in zebrafish could be divided into three successive steps: (i) AV canal (AVC) patterning; (ii) formation of endocardial cushions; (iii) remodeling of endocardial cushions into mature valve leaflets (Peal et al., 2011). During the heart looping and ballooning at ∼36 hpf, the atrium and ventricle are spatially separated by the AVC. Proper AVC development not only divides the heart into two chambers, but also builds the morphological milieu allowing subsequent formation of endocardial cushions and valves. At 48hpf, on each side of the AVC the single-layered early endocardial cushion cells have developed to acquire cuboidal shapes and express Alcam (activated leukocyte cell adhesion molecule). Eventually the endocardial cushions transform through cellular rearrangement into valve leaflets by 96 hpf (Beis, 2005; Peal et al., 2011; Pestel et al., 2016). Certain signaling pathways such as endocardial Notch (Timmerman et al., 2004) and myocardial Bmp (Ma, 2005) have been shown to be indispensable to ensure proper valvulogenesis. To monitor this process, several well-characterized valve marker genes were examined by WISH. During normal valvulogenesis, the flow-responsive gene *bmp4* gradually become stronger in myocardial cells in the AVC from 48hpf. At the same time, two other flow-dependent genes, *klf2a* and its downstream target *notch1b*, become progressively restricted to express in endocardial cells in the AVC. By 54 hpf, *klf2a* and *notch1b* are both significantly expressed within the endocardial cushions in the AVC, whereas in other domains of the endocardium their expression levels are lower or undetectable (Vermot et al., 2009; Donat et al., 2018). Nevertheless, in *vgll4b-*deficient embryos, the expression of *bmp4* and *notch1b* was misexpressed in the entire ventricle, whereas that of *klf2a* was almost disappeared in the AVC at 52 hpf (Figures 2H–J). All of these observations together with the absence of the cell migration marker *spp1* (Osteopontin or secreted phosphoprotein 1), a previously known factor that is upregulated in the AVC endocardial cushion in zebrafish (Peal et al., 2009), suggested the impairment of valvulogenesis (Figure 2K).

To further characterize the AVC defects in *vgll4b* mutant heart, we stained *vgll4b* mutant embryos with an Alcam-specific antibody (green) and phalloidin (red), which labels Actin within the entire heart. Compared with the wild type siblings, single confocal z-section images revealed that the endocardial cells within the AVC region were irregular shaped and disorganized in *vgll4b* mutants, further indicating that the valvulogenesis was affected due to the loss of *vgll4b* (Figures 2N,O).

All of these phenotypes could be recapitulated in the *vgll4b* morpholino (MO) antisense oligonucleotide injected morphants (data not shown). Specific *in vivo* rescue experiment was carried out and wild type *vgll4b* mRNA could effectively rescue all of the abnormalities, confirming the specificity of the phenotype observed.

### Disruption of Vgll4b-Mef2c Complex in Endocardium, Rather Than That of Vgll4b-Tead Complex, Is Responsible for the Defective Valvulogenesis Upon Loss of *vgll4b*

**FIGURE 3:**
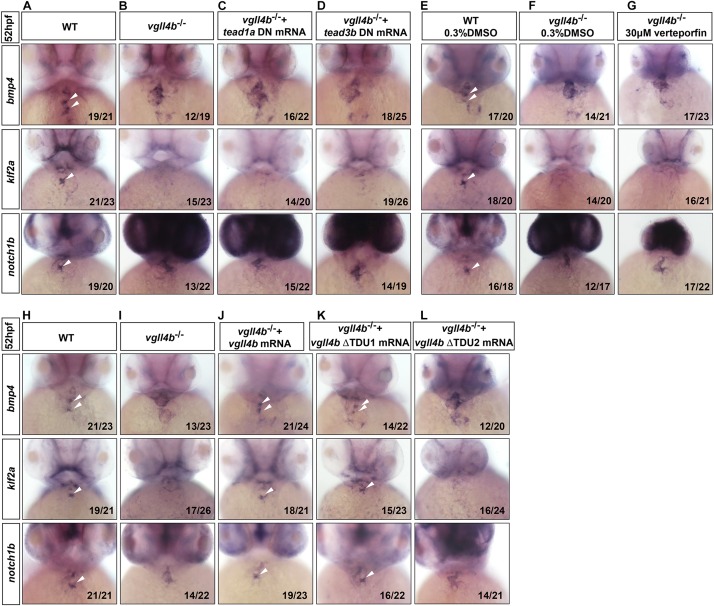
Tead is not involved in the impaired valvulogenesis in *vgll4b*-deficient embryos. **(A–D)** *Tead1a* and *tead3b* DN mRNA rescue assays in *vgll4b*^–/–^ embryos. *Bmp4*, *klf2a*, and *notch1b* probes were used in WISH. **(E–G)** *Vgll4b*^–/–^ embryos were treated with verteporfin (30 μM) from 32 hpf, but no rescue effect was observed. **(H–L)** *Vgll4b* mutants mRNA rescue assays in *vgll4b*^–/–^ embryos.

During the preparation of this manuscript, a research work showing that the murine VGLL4 also plays a role in valvulogenesis was published. Mechanistic studies indicate that endothelial VGLL4 controls valve development through negatively regulating the formation of TEAD-YAP complex (Yu et al., 2019). Nevertheless, it seems that similar mechanism is not utilized in zebrafish. In human genome, there are four *TEAD* genes known as *TEAD1* to *TEAD4*, whereas in zebrafish only *tead1* and *tead3* orthologs exist. However, the introduction of the dominant negative form of *tead1* or *tead3* (*tead* DN, in which the C-terminal Yap-binding domain was deleted) (Zhao et al., 2008) mRNA into *vgll4b*-deficient embryos displayed no rescue effects (Figures 3A–D). Similarly, treatment with verteporfin (a small molecule can interrupt Tead-Yap association) failed to rescue, either (Figures 3E–G). Moreover, it has been reported that TDU1 of VGLL4 preferentially interacts with TEF1 (TEAD1), whereas TDU2 tends to interact with MEF2 (Chen et al., 2004a). Yet, Vgll4b ΔTDU1 mutant mRNA displayed an effective rescue effect, whereas ΔTDU2 exhibited a much weaker rescue effect than that of ΔTDU1, suggesting that Mef2, rather than Tead, would be responsible for the impairment of valve development in zebrafish (Figures 3H–L and Supplementary Figure 1).

**FIGURE 4:**
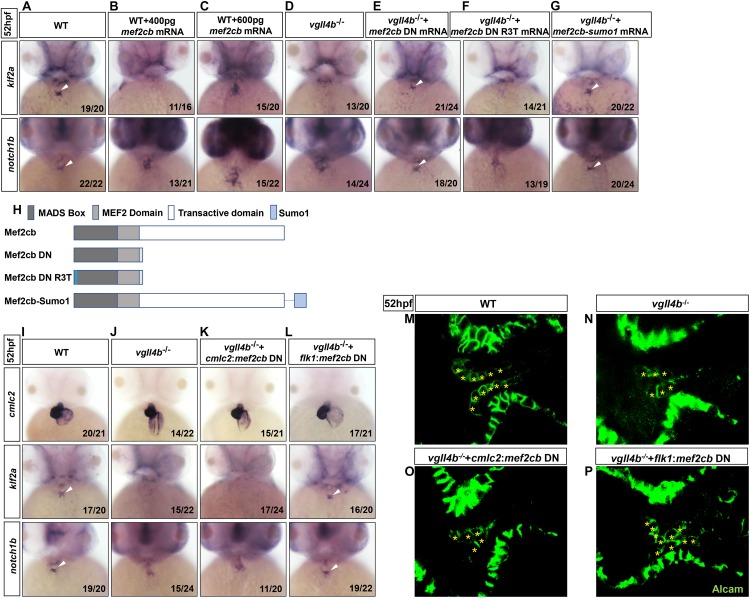
Aberrant activation of Mef2c due to the disruption of Vgll4b-Mef2c complex, accounts for the valvulogenesis defects in vgll4b mutants. **(A–C)** *Mef2cb* mRNA was injected into one-cell stage wild type embryos. **(D–G)** *Mef2cb* DN, *mef2cb* DN R3T, and *mef2cb-sumo1* mRNA rescue assays in *vgll4b*^–/–^ embryos. **(H)** Schematic representation of variant forms of Mef2cb, including WT, DN, R3T, and Sumo1 fusion mutants. **(I–L)** *Cmlc2*:*mef2cb* DN or *flk1*:*mef2cb* DN plasmid rescue assays in *vgll4b*^–/–^ embryos. **(M–P)** Representative images show Alcam staining of sibling and *vgll4b*^–/–^ embryos rescued with *cmlc2*:*mef2cb* DN or *flk1*:*mef2cb* DN plasmid at 52 hpf. The endocardial cushion cells expressing Alcam were indicated by asterisks. Scale bar: 10 μm.

In human or mouse, there are four conserved *MEF2* genes named *MEF2A* to *MEF2D*, of which *MEF2A* and *MEF2C* are enriched in heart and play a variety of functions during embryonic cardiac development and in adulthood (Chen et al., 1994; Naya et al., 2002; Durham et al., 2006; Huang et al., 2006; Ewen et al., 2011; Lockhart et al., 2013; Dong et al., 2017), thus Vgll4b might participate in cardiogenesis through a broader mechanism. Although the four *mef2* genes are all encoded in zebrafish genome, the main *mef2* genes expressed in the developing heart are *mef2a*, *mef2ca* and *mef2cb* (Hinits et al., 2012). The deficiency of *mef2a* leads to sarcomere assembly defects and severely impedes the cardiac contractility (Wang et al., 2005). *Mef2ca* and *mef2cb* are two alternatively spliced variants which play indispensable but redundant roles in myocardial cell differentiation (Ganassi et al., 2014). Among all of the MEF2 family members, MEF2C is the unique one which is expressed not only in cardiomyocytes, but also in endocardial cells to regulate valvulogenesis (Lockhart et al., 2013). Since in *vgll4b*-deficient embryos the most prominent abnormality was the defective valvulogenesis, we inferred that the Vgll4b-Mef2c complex might be disrupted upon loss of Vgll4b, and released free Mef2c protein in endocardial cells would impair valve development. To validate this hypothesis, firstly, *mef2cb* mRNA (400 pg) was introduced into one-cell stage wild type embryos, and the overexpression did phenocopy the aberrant expression patterns of *klf2a* and *notch1b* as observed in *vgll4b* mutants (Figures 4A,B,D). It’s worth noting that once 600 pg of *mef2cb* mRNA was introduced, an obvious misexpression of *klf2a* could be detected (Figure 4C). These results suggested that the severity of phenotype would be dose-dependent. Moreover, in *vgll4b* mutant embryos, the overexpression of *mef2cb* mRNA could cause more severe phenotype (data not shown). To demonstrate that Mef2c was overactivated, a dominant negative Mef2cb mutant mRNA (*mef2cb* DN) containing only the MADS and MEF2 domains (Molkentin et al., 1996; Ornatsky et al., 1997) was injected into *vgll4b* mutants, and a profound rescue effect emerged as expected (Figures 4E,H). To further demonstrate that the transcriptional activity of Mef2c was increased in endothelial cells, we put the same *mef2cb* DN gene under the control of *cmlc2* (*cmlc2*:*mef2cb* DN) or *flk1* (*flk1*:*mef2cb* DN) gene’s promoter (in Tol2 backbone) to limit its expression in cardiomyocytes or endothelial cells (Supplementary Figure 2). As anticipated, only the latter could restore the normal expression of *klf2a* and *notch1b*, as well as the pattern of the endocardial cells within the AVC region in *vgll4b*-defecient embryos (Figures 4I–P). It’s worth noting that the *flk1*:*mef2cb* DN construct could even rescue the morphological abnormality of the developing heart (Figures 4I–L), further indicating that the primary cause of the cardiac defects exists in endocardium.

MEF2C could not only function as a transcriptional activator but also a repressor through interaction with different cofactors which serve as positive or negative regulators of transcription (Dong et al., 2017). The post-translational modification SUMOylation mediates the MEF2-dependent repression (Gregoire and Yang, 2006; Kang et al., 2006; Riquelme et al., 2006). SUMO attachment to MEF2 occurs at a conserved lysine residue located in the C-terminal activation domain (Gregoire and Yang, 2006; Kang et al., 2006; Riquelme et al., 2006). Fusion of a SUMO1 molecule to the C-terminus of MEF2 turns it to be a repressor, which has been demonstrated for MEF2A, MEF2C, and MEF2D (Zhao et al., 2005; Gregoire and Yang, 2006; Kang et al., 2006; Shalizi et al., 2007). Hence, to clarify Mef2c functions as an activator or a repressor in valvulogenesis, *mef2cb-sumo1* fusion mRNA was used in rescue assay. The results showed that this repression form of Mef2c effectively restored the normal expression pattern of *klf2a* and *notch1b* in *vgll4b*-deficient embryos, suggesting Mef2c acts as an activator (Figures 4G,H).

MEF2 proteins can work by themselves or synergistically along with other transcription factor such as GATA4 (Morin, 2000). The MADS domain of MEF2 protein interacts with the zinc finger domain of GATA4, and the activation domain of both two proteins are indispensable for the functional synergy. However, the DNA binding capacity of MEF2 is not required (Morin, 2000). To elucidate whether Mef2c functions alone or through another transcription factor, a critical arginine which is necessary for DNA binding in MADS domain of Mef2cb (Mef2cb R3T) was mutated (Molkentin et al., 1996). We found that the rescue effect of this mutant was abolished, suggesting Mef2c directly binds to DNA (Figures 4F,H).

Overall, we conclude that in *vgll4b* mutants the increased free Mef2c protein in endocardium would affect the valvulogenesis through over-activating certain Mef2c downstream targets.

### Aberrant Activation of *klf2a* by Mef2c in Endocardium Contributes to the Impaired Valvulogenesis

**FIGURE 5:**
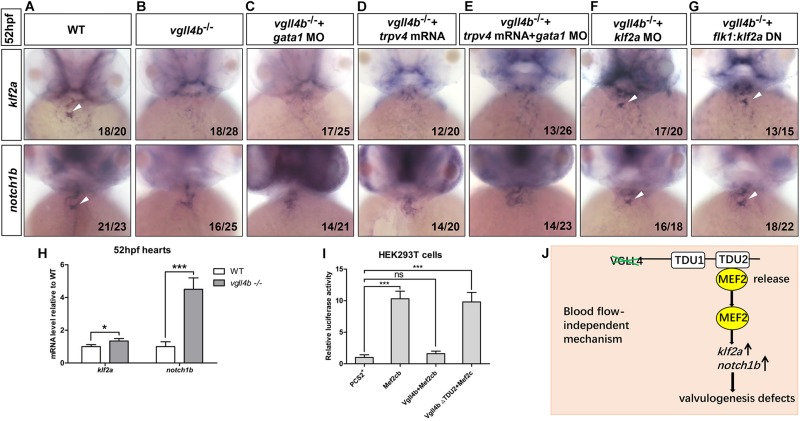
The failure of *klf2a* expression in the AVC is blood flow-independent, and *klf2a* is aberrantly activated by Mef2c in the endocardium, which ultimately impedes valvulogenesis. **(A–C)** *Vgll4b*^–/–^ embryos were treated with a high dose of *gata1* MO, but no rescue effect was observed. **(D,E)** *Trpv4* mRNA was injected alone or in combined with *gata1* MO into *vgll4b*^–/–^ embryos. **(F,G)** *Klf2a* MO and *flk1*:*klf2a* DN rescue assays in *vgll4b*^–/–^ embryos. **(H)** The hearts were harvested respectively from 35 wild type and 35 *vgll4b*^–/–^ embryos at 52∼54 hfp. Q-PCR analysis revealed that the expression level of *klf2a* and *notch1b* in *vgll4b*^–/–^ hearts was elevated (0.34-fold and 3.2-fold inductions compared to controls). Error bars represent ± SD of at least three replicates. *p* values are denoted by asterisks; ^∗^*P* < 0.1, ^∗∗∗^*P* < 0.001 (Student’s *t* test). **(I)** Dual luciferase vectors each with a fragment of the zebrafish *klf2a* promoter (–1.5 kb) were co-transfected into HEK293T cells with empty vector pCS2^+^, a *mef2cb* expressing vector, *mef2cb* combined with wild type *vgll4b* or *vgll4b* ΔTDU2 expressing vector. Luciferase activity was detected and normalized to empty vector pCS2^+^ which was set to 1.0. Error bars represent ± SD of at least three replicates. *p* values are denoted by asterisks; ^∗∗∗^*P* < 0.001 (ANOVA test). **(J)** Schematic depiction of the aberrant Vgll4b-Mef2c regulation in valvulogenesis in *vgll4b*^–/–^ zebrafish.

As the heart develops, the reversing (or oscillatory) flows between the ventricle and atrium becomes increasingly pronounced, which are most significant in the AVC precede valves formation (Vermot et al., 2009). The reversing flows have been shown to be an important factor in stimulating the expression of the flow-responsive genes *klf2a* and its downstream target *notch1b* in the AVC and guiding endocardial cell fate toward valvulogenesis (Vermot et al., 2009). As a key mediator of the effect of the reversing flows on valve development, once the reversing flows are reduced, *klf2a* is dramatically downregulated and subsequently affects valvulogenesis (Vermot et al., 2009). Injection of *gata1* (a gene controlling early hematopoiesis in zebrafish) MO can effectively increase the reversing flow fraction (RFF) through lowering blood viscosity (Vermot et al., 2009). Thus, to make sure whether the decreased *klf2a* expression in the AVC was caused by reduced RFF, we knocked down *gata1* gene in *vgll4b-*deficient embryos. However, even with a high dose of *gata1* MO depleting almost all circulating blood cells, no rescue effect of *klf2a* and *notch1b* could be found (Figures 5A–C). In addition, since it has also been reported that the endocardium senses the oscillatory flow via the mechanosensitive channel Trpv4 (transient receptor potential cation channel, subfamily V, member 4) to regulate the expression of *klf2a* (Heckel et al., 2015), *trpv4* mRNA was also introduced into *vgll4b*-deficient embryos. Nevertheless, neither *klf2a* nor *notch1b* could be rescued (Figure 5D). Moreover, the combined injection with *gata1* MO and *trpv4* mRNA had no obvious rescue effect, either (Figure 5E). Collectively, these results suggest that *vgll4b* deficiency leads to the decreased expression of *klf2a* in the AVC through a blood-flow-independent manner.

The cerebral cavernous malformation (CCM) pathway is necessary for cardiovascular development. CCM1 (CCM gene 1) and HEG1 (heart of glass) are two major components of the CCM protein complex, which inhibit the endocardial and endothelial expressions of *Klf2* during normal cardiovascular development in mice (Zhou et al., 2015, 2016). The loss of zebrafish Krit1 or Heg1 results in an increased expression of *klf2a* and *notch1b* throughout the endocardium in a blood-flow-independent manner, and as a consequence, the high levels of *klf2a* and Notch activity in endocardial cells ultimately impedes cardiac valve development (Donat et al., 2018). In our *vgll4b*-deficient embryos a drastic ectopic expression of *notch1b* did emerge at 52hpf, whereas no obvious misexpression of *klf2a* could be detected by WISH (Figures 3A,B). To evaluate more precisely the expression level of *klf2a*, the hearts were harvested from *vgll4b*-deficient embryos, and quantitative reverse transcription PCR (RT-qPCR) analyses were then performed. The results indicated that the transcription level of *klf2a* was even a little bit higher than that in the wild type siblings (Figure 5H), the discrepancy of the results obtained from the two methods could be explained by the low sensitivity of WISH to detect a weak increase of diffused signals in endocardium. Based on the observation that the activation of *klf2a* by Mef2cb is dose-dependent, we believe that the amount of released Mef2cb might not be enough to intensively activate *klf2a* transcription. To further demonstrate that the elevated expression of *klf2a* was the cause for the defective valvulogenesis, a mild dose of *klf2a* MO was injected, and a re-enrichment of both *klf2a* and *notch1b* in the AVC was observed (Figure 5F). Moreover, an *flk1* promoter driven dominant-negative *klf2a* DBD construct (*flk1*:*klf2a DN*, in Tol2 backbone) displayed a similar rescue effect (Figure 5G), confirming the relationship between the increased *klf2a* expression and valvulogenesis defects.

Next we set out to investigate the reason underlying the upregulation of *klf2a*. We mentioned above that in *vgll4b* mutants the released Mef2c would affect the valvulogenesis through over-activating certain downstream targets. It has been reported that MEF2 transcription factors can bind and transactivate *KLF2* promoter (Kumar et al., 2005). Furthermore, in endothelial cells the expression of *KLF2* was significantly activated by MEF2C (Xu et al., 2015). Hence, we speculated that the released Mef2c from Vgll4b-Mef2c complex would stimulate *klf2a* expression in *vgll4b* mutants. To test this hypothesis, a fragment of zebrafish *klf2a* promoter was cloned into a luciferase reporter vector, and the luciferase activity was increased when co-transfected with a *mef2cb* expressing plasmid in HEK293T cells. The results also showed that the ectopic expression of *vgll4b*, but not *vgll4b*ΔTDU2 effectively abolished the transcriptional activity of Mef2cb on *klf2a* promoter (Figure 5I).

Taken together, our findings indicate that the expression of *klf2a* is aberrantly activated by Mef2c upon loss of *vgll4b* in endocardial cells, which in turn impairs valvulogenesis (Figure 5J).

## Discussion

Heart valve defects are the most common causes of CHDs in approximately 2% of newborns (Hoffman and Kaplan, 2002). The cardiac gene regulatory network (GRN) contains a core set of evolutionary conserved transcription factors such as NKX2, MEF2, GATA, TBX, and HAND (Olson, 2006), along with multiple other regulators serving as accessory factors for these core transcription factors to contribute to cardiogenesis. To better understand the cardiogenesis, it is important to identify and characterize the novel regulators in this process. Among all of the transcription cofactor *VGLL* family members, *VGLL4* is the only one expressed in human heart (Chen et al., 2004a). Interestingly, *VGLL4* is located within the CHD sensitive region of 3p25 deletion syndrome which is frequently related to atrioventricular septal defect (AVSD) (OMIM: 613792), highlighting its role in valvulogenesis.

The genes involved in Notch and Bmp pathways must get spatiotemporally from heart-wide to AVC-specific to set up endocardial cushion formation. Although reversing flows have been shown to play a critical role in this process (Vermot et al., 2009), the mechanism directing such restriction is still poorly understood. Therefore, it is important to define the molecular details controlling AVC patterning and subsequent valve development in order to gain further insights toward the etiology of valve diseases. In this study, we identified a pivotal role of Vgll4b in controlling valvulogenesis through Mef2c sequestration. As a consequence of *vgll4b* deficiency, the Mef2c target *klf2a* is activated, which ultimately impairs valve development. Thus the mechanism governing the restricted expression of these flow-responsive genes in the AVC is not merely stimulation by blood flow, but the repression throughout the endocardium by certain regulators. In addition, although altered expression patterns were observed for *bmp4*, *klf2a*, and *notch1b* in the AVC in *vgll4b*-deficient mutants, some other valve markers such as *tbx2b* and *has2* (hyaluronan synthase 2) kept intact (data not shown). Transcription factor Tbx5 has been shown to restrict the expression of *tbx2b* and *has2* in the AVC to elicit the formation of the endocardial cushion and valves (Camarata et al., 2010). These observations suggested that the valvulogenesis is a very sophisticated process in which retrograde blood flow and multiple regulators function coordinately to ensure proper formation of valves.

Although defective heart valve development was observed in both *Vgll4*-deficient mice and *vgll4b*-deficient zebrafish, different mechanism seems to be implicated. While imbalance of VGLL4-TEAD and TEAD-YAP complexes in Hippo pathway is responsible for valvulogenesis defects in mice (Yu et al., 2019), abnormal expression of *klf2a* in endocardium caused by disruption of Vgll4b-Mef2c complex impairs the valvulogenesis in zebrafish. Beyond that, since there is only one wildly expressed *Vgll4* gene exists in mice, whereas three *vgll4* paralog genes in zebrafish, more differences were observed. For example, the size of *Vgll4*^–/–^ mice is much more less than that of siblings, but such phenomenon could not be found in *vgll4b*^–/–^ zebrafish (data not shown). Moreover, a majority of *Vgll4*^–/–^ mice died shortly after birth (Yu et al., 2019), whereas the *vgll4b*-deficient zebrafish could survive into adulthood. In addition, an incomplete penetrance (∼65%) was found for both *vgll4b*^–/–^ and *vgll4b* morphants. These differences would be explained by the compensation of *vgll4a* and/or *vgll4l* paralogs in zebrafish.

The relationship between VGLL4 and its interacting proteins is intriguing. On one side, the acetylation of VGLL4 at lysine 225 in TDU1 can regulate TEAD1 degradation through a cysteine protease-dependent pathway (Lin et al., 2016). On the other side, VGLL4 can interact with IRF2BP2 independent of TDU domains and enhance its protein stability through preventing proteasome-mediated degradation (Wu et al., 2019). Nevertheless, the protein level of Mef2cb was not affected in HEK293T cells co-transfected with Vgll4b (data not shown). Thus the possibility that Vgll4b controlling the stability of Mef2cb could be excluded.

Most partners of VGLL4 are unable to induce transcription on their own. For example, TEAD proteins have to interact with transcriptional cofactors to activate transcription (Vassilev et al., 2001; Pobbati et al., 2012); IRF2BP2 is frequently described as a corepressor (Childs and Goodbourn, 2003; Carneiro et al., 2011). We thus believe that VGLL4 might be a reservoir of these transcription regulators, once Vgll4 is mutated, its partners would be released and lead to pathogenesis.

## Data Availability Statement

All datasets generated for this study are included in the article/Supplementary  Material.

## Ethics Statement

The animal study was reviewed and approved by Animal Ethical and Welfare Committee, Rui-Jin Hospital, Shanghai Jiao Tong University School of Medicine, Shanghai, China.

## Author Contributions

CX, XL, BW, and SG performed the experiments and analyzed the data. RY established the *vgll4b* knockout line. JT and JZ participated in the preparation of the manuscript. JZ designed the research, analyzed the data, and wrote the manuscript.

## Conflict of Interest

The authors declare that the research was conducted in the absence of any commercial or financial relationships that could be construed as a potential conflict of interest.

## Footnotes

1 http://zifit.partners.org/ZiFiT
