# Supplementary material for: Zebrafish Vestigial Like Family Member 4b Is Required for Valvulogenesis Through Sequestration of Transcription Factor Myocyte Enhancer Factor 2c
Source: Front Cell Dev Biol. 2019 Nov 12;7:277. doi: 10.3389/fcell.2019.00277 (PMC6874126; doi:10.3389/fcell.2019.00277)
Supplement: Supplementary file 1 [file Data_Sheet_1.PDF]

Supplemental figures

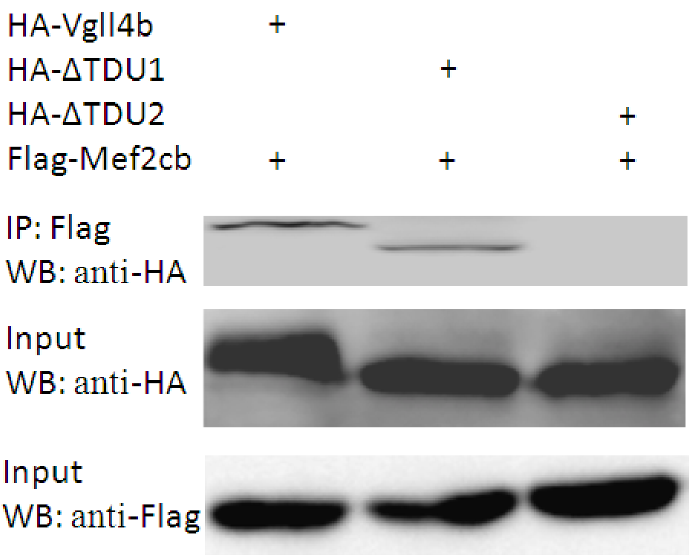

Figure 1: Wild type Vgll4b and Vgll4b ΔTDU1 mutant, but not the ΔTDU2 mutant, was immunoprecipitated (IP) from HEK293T cells coexpressing Mef2cb.

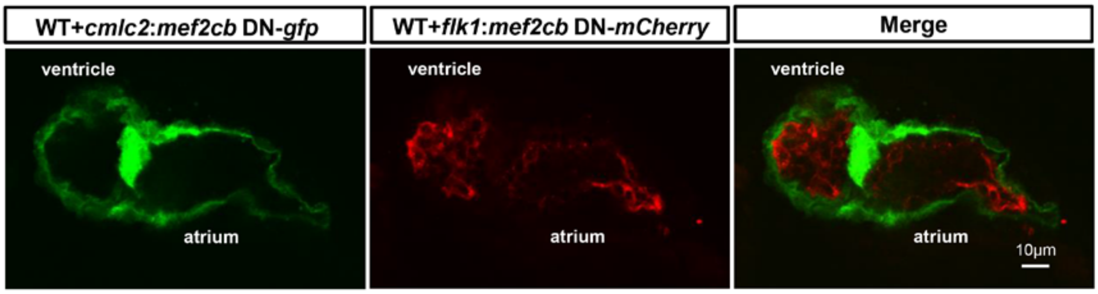

Figure 2: *Cmlc2*:*mef2cb* DN-*gfp* and *flk1*:*mef2cb* DN-*mCherry* plasmids (both in Tol2 backbone) were transiently expressed in zebrafish embryos by co-injecting 80 pg of Tol2-plasmids and 120 pg of Tol2 transpose mRNA at one-cell stage. At 52hpf, the signal of GFP could be specifically observed in myocardium, whereas that of mCherry was found only in endocardium.
